# Severe Paediatric Trauma in Australia: A 5-Year Retrospective Epidemiological Analysis of High-Severity Fractures in Rural New South Wales

**DOI:** 10.3390/jcm14144868

**Published:** 2025-07-09

**Authors:** David Leonard Mostofi Zadeh Haghighi, Milos Spasojevic, Anthony Brown

**Affiliations:** 1 Sydney Medical School, The University of Sydney, Sydney, NSW 2050, Australia; 2 Department of Orthopaedic Surgery, Townsville University Hospital, Townsville, QLD 4814, Australia; 3 Faculty of Medicine, The University of Queensland, Brisbane, QLD 4006, Australia; 4 Department of Orthopaedics, Traumatology and Sports Medicine, Spital STS AG, 3600 Thun, Switzerland; 5 School of Rural Health, The University of Sydney, Dubbo, NSW 2830, Australia

**Keywords:** paediatric, traumatology, fracture, epidemiology, rural

## Abstract

**Background**: Trauma-related injuries are among the most common reasons for paediatric hospital presentations and represent a substantial component of orthopaedic care. Their management poses unique challenges due to ongoing skeletal development in children. While most reported fractures occur at home or during sports, prior studies have primarily used data from urban European populations, limiting the relevance of their findings for rural and regional settings. Urban-centred research often informs public healthcare guidelines, treatment algorithms, and infrastructure planning, introducing a bias when findings are generalised outside of metropolitan populations. This study addresses that gap by analysing fracture data from two rural trauma centres in New South Wales, Australia. This study assesses paediatric fractures resulting from severe injury mechanisms in rural areas, identifying common fracture types, underlying mechanisms, and treatment approaches to highlight differences in demographics. These findings aim to cast a light on healthcare challenges that regional areas face and to improve the overall cultural safety of children who live and grow up outside of the metropolitan trauma networks. **Methods**: We analysed data from two major rural referral hospitals in New South Wales (NSW) for paediatric injuries presenting between 1 January 2018 and 31 December 2022. This study included 150 patients presenting with fractures following severe mechanisms of injury, triaged into Australasian Triage Scale (ATS) categories 1 and 2 upon initial presentation. **Results**: A total of 150 severe fractures were identified, primarily affecting the upper and lower limbs. Males presented more frequently than females, and children aged 10–14 years old were most commonly affected. High-energy trauma from motorcycle (dirt bike) accidents was the leading mechanism of injury among all patients, and accounted for >50% of injuries among 10–14-year-old patients. The most common fractures sustained in these events were upper limb fractures, notably of the clavicle (n = 26, 17.3%) and combined radius/ulna fractures (n = 26, 17.3%). **Conclusions**: Paediatric trauma in regional Australia presents a unique and under-reported challenge, with high-energy injuries frequently linked to unregulated underage dirt bike use. Unlike urban centres where low-energy mechanisms dominate, rural areas require targeted prevention strategies. While most cases were appropriately managed locally, some were transferred to tertiary centres. These findings lay the groundwork for multi-centre research, and support the need for region-specific policy reform in the form of improved formal injury surveillance, injury prevention initiatives, and the regulation of under-aged off-road vehicular usage.

## 1. Introduction

Paediatric orthopaedic emergencies represent a significant proportion of paediatric presentations to emergency departments and orthopaedic services worldwide. These injuries arise from various causes, with the most common being falls in the home or playground accidents, as well as less frequent high-velocity trauma and assaults [1]. Certain fracture patterns are frequently observed in specific age groups. Distal forearm and clavicle fractures, for example, are among the most commonly encountered fractures in children, whereas femoral shaft fractures are relatively rare [2]. Outdoor and sports-related injuries, along with falls, are universally recognised as the primary mechanisms of injury among children, and non-operative management is more commonly employed than surgical intervention in the treatment of paediatric fractures [3].

In rural settings, the mechanisms of injury and associated fracture patterns often differ from those observed in urban areas [4]. Although there is limited literature specifically addressing this issue in the paediatric population, trends have been noted in adult trauma. In rural areas, there is a higher incidence of injuries related to recreational activities such as motorbike and horse riding, as well as incidents involving farm machinery and high-speed vehicle collisions [5]. This difference in injury patterns necessitates the need for a better understanding of how rural environments and lifestyle factors contribute to paediatric traumatology.

Children with fractures constitute a large cohort of presentations to emergency departments in both rural and urban settings [6]. The management of these injuries is often complicated by the ongoing skeletal development of the child and the emotional distress experienced by both the patient and their family. One significant complication in paediatric orthopaedics is the disruption of the growth plate (physis) during surgical intervention, which can result in growth arrest, a major long-term concern [7]. Furthermore, the emotional and logistical burdens on families are particularly acute in rural areas, where distances between home and hospital can be vast. This is exacerbated when a child requires transfer to a tertiary centre, for specialised treatment, especially when emergency retrieval is necessary, and family members cannot immediately accompany the patient [8]. This highlights the importance of optimising both medical and logistical care for children in rural settings, with the objective to reduce the long-term impacts of these injuries.

Despite the frequency of these presentations, there remains a gap in the literature regarding the epidemiology of paediatric fractures in rural areas. Most studies have focused on specific fracture types, such as supracondylar fractures, and their associated outcomes, rather than providing comprehensive data on fracture patterns in children as a whole, although some studies have evaluated fracture patterns, mechanisms of injury, and outcomes in metropolitan settings [1,2,3]. There is limited research that comprehensively addresses the epidemiology of paediatric traumatology in rural populations. A Swedish study, which included both metropolitan and rural areas, evaluated 9965 paediatric patients but did not specifically focus on rural-specific fracture patterns [9]. The need for this research is clear. The lack of accessibility to paediatric trauma registries outlines a research gap, in a field where the mortality of children in rural areas is up to 2.4 times greater following injury than that of children in urban areas [10]. Understanding specific injury patterns in rural populations is critical for developing effective preventative strategies, as well as improving cultural safety (a term referring to care that is responsive to the cultural identity and needs of patients, as defined by those receiving the care) for rural children by tailoring medical interventions to local needs [11].

This study aims to fill this gap by providing a comprehensive analysis of severe fracture presentations in rural children, identifying the mechanisms of injury, and examining the treatments most commonly associated with these fractures. By offering insights into these factors, this study aims to inform clinical practice, improve patient outcomes, and guide the development of tailored preventative and treatment strategies that address the unique challenges faced by rural populations. Ultimately, this research will contribute to enhancing the quality of paediatric orthopaedic care, particularly in underserved rural areas, ensuring that children receive timely and appropriate interventions, regardless of their geographic location.

## 2. Materials and Methods

### 2.1. Design

This study uses a retrospective cross-sectional design, focusing on paediatric patients (<18 years of age) who presented to the emergency departments of two regional trauma centres in rural New South Wales between 1 January 2018 and 31 December 2022. The collected data was analysed to generate a comprehensive descriptive dataset, highlighting key demographic characteristics and providing insights into the prevalence of various fracture types within this population.

### 2.2. Data Source

Following approval from the Greater Western Human Research Ethics Committee (approval number: 2021/ETH11791), data were extracted from the Emergency Department dataset by the Health Information Unit (HIU) of the Western NSW Local Health District. Initially, data covering the period from 1 July 2017 to 13 December 2021 were provided, with additional data extracted for the period from 13 December 2021 to 21 December 2022. The datasets included all paediatric (<18 years of age) presentations to two separate rural trauma centre emergency departments for acute injuries. Variables included age, date and time of presentation, sex, suburb or town, presenting problem, ATS classification, and diagnosis description. The medical record number (MRN) was also provided, enabling access to further information from the electronic Medical Record (eMR).

**Figure 1:**
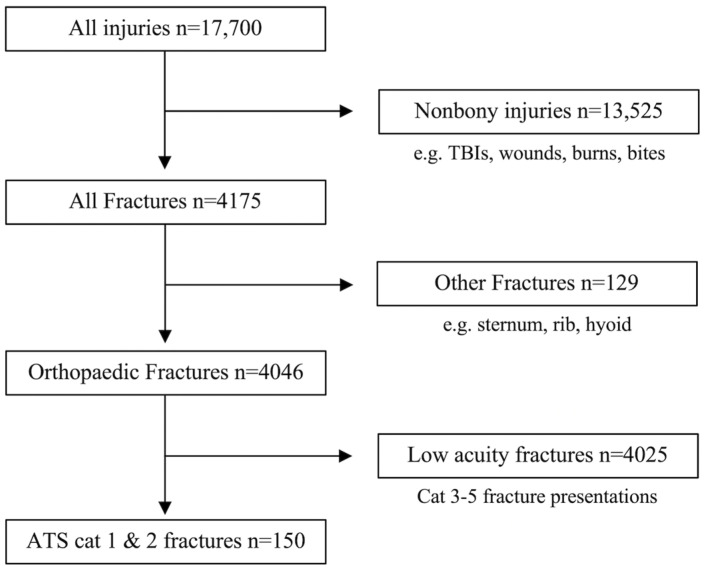
The flow chart outlining the inclusion and exclusion processes to achieve a group of l50 severe injuries from the initial database of all presentations to both hospitals from January 2018 to December 2022.

The two datasets were combined, duplicates were excluded, and records outside the study period (January 2018 to December 2022) were removed, resulting in five years’ worth of data and 17,700 injury records. Presentations associated with a “fracture” were identified using the presenting problem and diagnosis description codes. The dataset was then filtered to include only fractures typically managed by the orthopaedic and trauma surgery team, specifically fractures of the long bones, spinal column, scapula, clavicle, and pelvis. Fractures involving the face, nose, cranium, ribs, hyoid, or sternum were excluded. The Australian Triage System (ATS) is the standardised triage tool used in emergency departments across Australia. Each included an ATS classification (1 to 5, with cat 1 being most urgent). The dataset was filtered to include only high-acuity cases, and cat 3–5 presentations were excluded from analysis. The resulting final group of 150 severe presentations was included in this study for analysis (Figure 1).

The 150 patients with high-severity fractures were further examined in the electronic Medical Record (eMR) system of the Western NSW Local Health District to capture details on mechanism of injury, type of treatment, and outcome. The final dataset incorporated the following variables:

From the HIU dataset: age, date and time of presentation, sex, suburb or town, presenting problem, and diagnosis description.

From the eMR system: mechanism of injury, type of treatment, and outcome.

There were no records with missing key clinical or demographic variables identified (e.g., presenting problem, mechanism of injury, fracture type, treatment method, etc.) that required exclusion from analysis.

### 2.3. Data Safety

All data associated with this study are securely stored in an encrypted database at the University of Sydney, located on the Camperdown campus. Access is restricted to authorised personnel via a VPN, which securely connects to the university’s mainframe. Data cannot be transferred or stored on personal devices, ensuring compliance with privacy and security protocols.

### 2.4. Ethics

This study was approved by the Greater Western Human Research Ethics Committee and classified as posing a low or negligible risk.

## 3. Results

### 3.1. Combined Results

**Figure 2:**
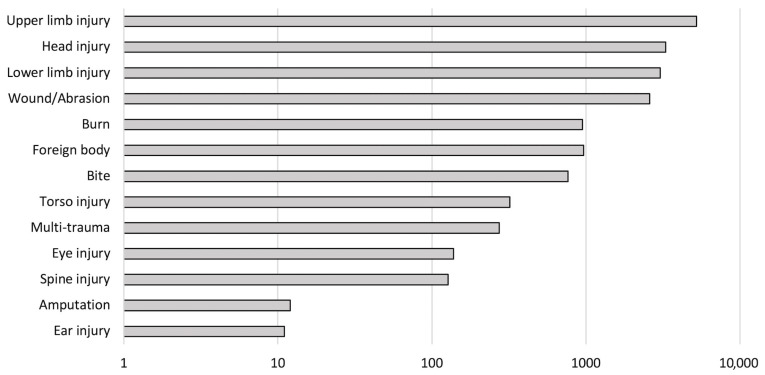
Number of presentations, categorised by presenting complaint (log scale), presenting to both hospitals.

Between January 2018 and December 2022, there were 17,700 injury-related presentations to the emergency departments of two regional trauma centres. The majority of these cases involved orthopaedic injuries, including upper and lower limb fractures, soft tissue injuries, muscular injuries, spinal injuries, amputations, and wounds. Undifferentiated upper limb injuries (encompassing both soft tissue and bony injuries) represented 29.5% (n = 5229) of all injuries, while undifferentiated lower limb injuries accounted for 17.1% (n = 3034). Other injury types included burns, soft tissue injuries, head injuries, and multi-traumas (Figure 2). Notably, amputations, although typically restricted to the limbs, were coded separately, preventing the identification of the precise amputation site.

A total of 9122 (51.5%) presentations were identified as potentially requiring orthopaedic intervention, including lacerations, amputations, fractures, and foreign bodies in limbs. Among these, 4046 (22.9%) were fractures. Non-bony injuries and fractures categorized under triage categories 3–5 were excluded. On initial triage, 12% (n = 18) of patients were classified as category 1 (immediate attention), while the remaining 88% (n = 133) were classified as category 2 (urgent attention).

Demographic data for these 150 high-severity presentations are summarised in Table 1. The gender distribution was predominantly male, with 113 (75.3%) male patients and 38 (24.7%) female patients. The largest age group was 10–14 years (42.7%), followed by 5–9 years (25.3%) and 15–19 years (26.0%). Only 6.0% of presentations were from children aged 0–4. Patients arrived by air ambulance (4.0%, n = 6), road ambulance (48.0%, n = 72), or private transport (47.3%, n = 71).

Fractures were categorised into limb fractures (Table 2) and “other” fractures (including spinal, pelvic, and extremity fractures) (Table 3). Upper limb fractures constituted 84.0% (n = 126) of all fractures, with 117 (78%) being closed fractures and 11 (7.3%) being open fractures. The most frequent fractures were clavicle fractures and combined radius and ulna fractures, making up 17.3% (n = 26) of injuries. The midshaft clavicle was the most commonly fractured site (13.3%, n = 20), followed by the distal radius and ulna (10.0%, n = 15). Open fractures included three radius and ulna fractures (2%) and one humerus fracture (0.7%).

Lower limb fractures accounted for 26% (n = 39) of fractures, with 34 (22.7%) being closed and 5 (3.3%) being open fractures. Femoral fractures were the most common (10%, n = 15), followed by tibia fractures (6.7%, n = 10) and combined tibia and fibula fractures (4.7%, n = 7). Isolated fibula fractures were rare (1.3%, n = 2). In femur and tibia fractures, the midshaft was the most commonly fractured site, representing 7.3% (n = 11) and 4% (n = 6) of fractures, respectively. Combined open tibia and fibula fractures were the most frequent open fractures in the lower limbs (2.7%, n = 4), with an even split between midshaft and distal fractures.

Other less common fractures included spinal column, pelvic, and extremity fractures, comprising 16% (n = 24) of all fractures. Spinal fractures were the most frequent outside of the limbs, accounting for 7.3% (n = 11) of total fractures, including three cervical spine fractures (2%), five thoracic spine fractures (3.3%), one lumbar spine fracture (0.7%), and two sacral fractures (1.3%). Additionally, six fractures involved the hand (4%), including one open phalanx fracture (0.7%), five foot fractures (3.3%), and two pelvic fractures (1.3%).

Table 4 displays the distribution of injuries by age group and mechanism of injury, highlighting that 40.7% of all injuries across age groups were due to motorbike accidents. These findings highlight the significant overall burden associated with motorbike-related trauma, with the next most common mechanism, falls, accounting for just 18.6% of injuries, less than half the proportion attributed to motorbike incidents. Notably, in the 10–14-year age group, over half of the presentations (51.6%) were due to motorbike-related injuries.

Table 5 outlines the common mechanisms of injury for each fracture type. In most cases, a single mechanism predominated for each fracture type, with exceptions for isolated ulna, fibula, and hand fractures. Motorbike accidents (MBAs) were the most common mechanism for six fracture types, particularly those involving the spine, radius, and clavicle, where MBA-related injuries constituted the majority (>50%) of these fractures. Other frequent mechanisms included playground-related falls, which were most often associated with upper limb fractures (humerus and radius/ulna fractures), and equestrian accidents, which commonly caused humeral fractures.

Table 6 presents the treatment modalities for various fractures. Non-operative management involved pain relief, immobilisation with a sling, cast, splint, limb traction, or close follow-up. Closed reduction and casting (CR) were the most common treatments for isolated radius, ulna, radius/ulna, and tibia/fibula fractures, while femoral fractures were most frequently treated with intramedullary nailing (IM nail). Other treatment methods included closed reduction with K-wire fixation (CR + KW), open reduction with internal fixation (ORIF), or transfer to specialist centres such as Westmead Children’s Hospital (n = 5) or the Royal North Shore Hospital spinal unit (n = 1). In total, 46.7% (n = 70) of patients received conservative treatment, 31.3% (n = 47) underwent closed reduction with or without fixation, 11.3% (n = 17) received ORIF, 8.0% (n = 12) underwent IM nailing, and 4.0% (n = 6) were transferred to a specialist tertiary service. Additionally, 3.3% (n = 5) were retrieved for emergency spine and pelvis surgery, and 0.7% (n = 1) was referred for revision surgery after delayed union, following initial closed reduction and percutaneous K-wire pinning.

#### 3.1.1. Hospital 1 Cohort

Of the total patients included in this study, 46.7% (n = 70) presented to the emergency department of Hospital 1. The demographic information for these patients is summarised in Table 2. The gender distribution was predominantly male, with 78.6% (n = 55) males and 21.4% (n = 15) females. The age distribution mirrored that, of the overall cohort, the largest group was 10–14 years (n = 33, 47.1%), followed by 5–9 years (n = 19, 27.1%), 15–19 years (n = 15, 21.4%), and 0–4 years (n = 3, 4.3%). All patients at Hospital 1 were categorised under triage category 2 (emergency), with the primary modes of arrival being private car (n = 36, 51.4%), road ambulance (n = 33, 47.1%), and air ambulance helicopter (n = 1, 1.4%).

#### 3.1.2. Hospital 2 Cohort

Of the total number of patients included in this study, 53.3% (n = 80) presented to the emergency department of Hospital 2. Demographic information is summarised in Table 2. The cohort consisted of 58 males (72.5%) and 22 females (27.5%). The age distribution mirrored that, of the patients presenting to Hospital 1, the largest group was 10–14 years (38.8%, n = 31), followed by 15–19 years (30.0%, n = 24), 5–9 years (23.8%, n = 19), and 0–4 years (7.5%, n = 6). Patients at Hospital 2 were categorised into both triage categories 1 (resuscitation) and 2 (emergency), receiving 100% of the patients classified into category 1 in this study, representing 22.5% (n = 18) of their total presentations. The remaining 77.5% (n = 62) were triaged as category 2.

The predominant mode of arrival to Hospital 2 was by road ambulance (48.8%, n = 39), followed by private transport (43.8%, n = 35) and air ambulance helicopter (6.3%, n = 5). Notably, the frequency of air ambulance arrivals to Hospital 2 was three times higher than that of Hospital 1 during the same period. This can be attributed to Hospital 2′s role as the regional trauma service (RTS) during the study period, and would further explain receiving all category 1 presentations.

## 4. Discussion

Fractures are a common occurrence in both the paediatric and elderly populations, with paediatric fractures primarily attributed to bone immaturity [2]. This study is particularly significant as it demonstrates clear patterns in paediatric fractures following severe injuries in a rural setting, offering valuable insights into the unique injury mechanisms and fracture types prevalent in these populations. Our findings reveal that, among children presenting to rural emergency departments in NSW, Australia, upper limb fractures, particularly clavicle fractures and combined radius and ulna fractures, were the most common, predominantly resulting from high-energy mechanisms. Motorcycle (dirt bike) accidents accounted for more than 50% of presentations involving fractures of the spine, clavicle, and radius.

The epidemiology of fractures in children has been extensively studied [1,2,3,9], but much of the existing research is conducted in metropolitan hospitals, where injury patterns and mechanisms differ significantly from those in rural areas. Previous studies identified distal forearm fractures as the most common injury in paediatric trauma [1,2,3]. Our study corroborates these findings, where distal forearm fractures account for 18.5% of all fractures. A key distinction in our cohort is the greater contribution to injury causation from high-energy mechanisms. By highlighting injury severity and mechanism in regional populations, our findings aim to inform healthcare policy interventions focused on awareness, prevention, and surveillance system improvement, particularly in settings with diverse cultural needs.

Three previous studies [1,2,3] concluded that falls were the most common mechanism of injury in infants and young children. Falls accounted for as high as 54% of injuries in the study by Hedström et al., while they noted sport-related injuries and traffic collisions increased in prevalence with age [3]. Our findings diverge from these studies, as we identified motorbike accidents as the predominant cause of injury, particularly in the 10–14-year age group, where they account for 51.6% of all fractures. To highlight additional but isolated instances of high-energy mechanisms causing severe traumatic injuries in this study, one child caught their arm in a tractor shaft and sustained open fractures of both the radius and ulna with an elbow dislocation; a water-skiing collision with a tree lead to one child sustaining closed unilateral proximal tibia and fibula fractures; and a dive into a shallow body of water caused C2–3 and C7 neck fractures for one child. These findings not only outline the contrast in the severity of injury mechanisms between regional and urban paediatric populations, but the notable burden of injury associated with unregulated motorbike riding in regional areas, posing the question, why are young children on motorbikes and what is being done to protect them? Region-specific research is required to better understand the epidemiology of fractures in rural paediatric populations and for targeted interventions, aimed at reducing preventable traumas to begin with.

The age and gender distribution in this study aligns with previous research [1,2,3]. Our study also found a higher incidence of fractures in males of all age groups, but particularly in the 10–14-year age group. This is consistent with Hedström et al.’s findings, which indicated that males in rural areas are at a greater risk of sustaining physical trauma [3]. The increased involvement of young males in high-risk recreational activities and risk-taking behaviours likely accounts for this finding [3]. Furthermore, in a study examining fracture presentations in both metropolitan and rural areas, Hedström et al. noted that living in a rural environment appeared to be a protective factor against sustaining fractures, based on comparisons with a metropolitan cohort [9], a finding which was supported by Gilbride et al. [12]. However, these results contrast with those of four separate studies, which reported a higher incidence of self-reported, medically attended, and fatal injuries in rural children [13,14,15,16]. To further understand these discrepancies, an analysis of the underlying factors contributing to the differences in injury rates between rural and metropolitan populations is required, taking into account socioeconomic, environmental, and healthcare accessibility variables.

Motorbike riding has previously been identified as a risk for children in rural and remote areas. Mulligan et al. conducted a study of paediatric off-road vehicle injuries in rural Australia and found patterns of injuries occurring despite high rates of protective gear use [17]. They recommend further investigation into the injury mitigation and fit properties of protective gear [17]. There are currently no restrictions on motorcycle riding on private properties in Australia, with many children engaging in both recreational dirt bike riding and as a mode of transport around large properties.

Toida et al. conducted a study on paediatric injury trends in Japan [18]. They highlighted the importance of enhanced injury surveillance systems to guide the development of targeted injury prevention strategies and evaluate care quality. Building on their findings, it is clear that there is a necessity for similar improvements in injury surveillance and prevention strategies in rural Australia. Additionally, the ongoing evaluation of the quality of injury care, as recommended by Toida et al., would help ensure that healthcare services in rural regions are equipped to effectively manage the high morbidity resulting from preventable trauma [18]. Our findings additionally suggest the need for policy-level improvements, in the form of more systematic and standardised paediatric injury surveillance across New South Wales and Australia. The state of Victoria, through the Victorian Injury Surveillance Unit at Monash University, has established itself as a national leader in this area, providing a model for comprehensive data collection and utilisation [19]. In contrast, current systems in NSW are limited in scope. The Institute of Trauma and Injury Management (ITIM) collects data on major trauma presentations, contributing to the Australian Trauma Quality Improvement Program (AusTQIP) and the Australian Trauma Registry (ATR) [20]. However, the paediatric trauma data captured by ITIM are drawn exclusively from the three level 1 paediatric trauma centres in NSW—Sydney Children’s Hospital, Westmead Children’s Hospital, and John Hunter Children’s Hospital [21]. This approach fails to adequately represent rural trauma presentations and obscures potential disparities between urban and rural paediatric trauma presentations in the ITIM’s annual reports. A more inclusive and geographically comprehensive surveillance framework, similar to that which already exists for Neurotrauma in rural NSW, is essential to inform targeted injury prevention strategies and guide public health policy development at both the state and national levels [22]. The establishment of such systems would not only improve patient outcomes but also allow for data-driven policy decisions to reduce the long-term healthcare burden in regional communities.

This study was not without its limitations. The reliance on the electronic Medical Record (eMR) system to extract key variables such as mechanism of injury and treatment introduced inherent constraints, particularly regarding follow-up data. The patients who required transfer to tertiary centres for definitive management were not fully accounted for, resulting in gaps in the dataset and a loss of follow-up notes (outcome variables). Consequently, while this study effectively captures initial presentation patterns and triage classifications at the local hospital, it does not comprehensively assess long-term outcomes or, more importantly, the impact of time to surgery by inter-hospital transfer on clinical outcomes. Furthermore, the use of the Australasian Triage Scale (ATS) to identify severe injuries introduced potential limitations. While triage categories 1 and 2 are indicative of need for urgent medical attention, they do not necessarily correlate with the actual severity of injury. In some cases, patients were triaged into high-acuity categories based on their mechanism of injury, in anticipation of a severe polytrauma, which later did not correlate with the actual severity of their skeletal trauma following the primary survey. For example, high-speed dirt bike accidents frequently triggered trauma team activation; however, some of these cases later revealed single isolated injuries only. Refined tools that better delineate true injury severity, such as a scoring criteria following the primary survey of polytrauma patients, would provide a more accurate measure of true injury severity for categorising patients.

Inconsistencies in database coding further complicated data extraction and analysis. Variability in fracture classification, ranging from non-specific labels such as “fracture of bone” to anatomically precise terms such as “fracture of distal radius”, posed challenges in standardising data interpretation. Additionally, errors in anatomical coding were later identified in some cases when reviewing the eMR, emphasising the necessity for a more rigorous and standardised injury classification framework. This would enhance data reliability, facilitating more accurate epidemiological analyses in future.

The relatively small sample size of this study also limited its statistical power, precluding robust inferential analysis. While the observed trends provide valuable insights into the fracture patterns and injury mechanisms prevalent in rural paediatric populations, a larger, multi-centre study, including both rural trauma centres and metropolitan level 1 trauma centres, would be required to determine statistical significance and generalisability. Future research should incorporate broader datasets across multiple rural and metropolitan hospitals to enable meaningful comparisons and strengthen epidemiological conclusions, including the impact of transfer times on outcomes for those patients who required transfer for specialist services [23]. The social determinants of outcomes that require additional consideration, but were not collected during this study, include geographical isolation from the nearest hospital capable of handling trauma, which may often be hundreds of kilometres in Australia, as well as the socioeconomic factors involved in travel and prolonged time off work to accompany a child to a major city.

Despite its limitations, this study offers novel contributions to the understanding of paediatric traumatology in rural settings. The predominance of high-energy trauma mechanisms, particularly motorbike-related injuries, highlight the unique injury landscape of non-urban populations, contrasting sharply with the fall- and sport-related fractures that were more commonly reported in previous metropolitan studies. While the rural perspective that has been relatively under-represented in prior research, by delineating the distinct injury mechanisms and fracture patterns in this population, our findings serve as a foundation for future studies and may inform clinical practice and public health initiatives aimed at reducing the burden of paediatric trauma in rural communities. Further multi-centre research, incorporating larger cohorts and standardised data collection methodologies, will be essential in refining local trauma care strategies and optimising paediatric trauma management across diverse geographical settings, with the overall aim of improving cultural safety for children and families living outside the immediate reach of metropolitan trauma networks.

## 5. Conclusions

Paediatric trauma remains a significant public health challenge in regional Australia. This study highlights the distinct and under-reported consequences of large-scale unregulated motorcycle use, where the high-energy trauma demographic is closely linked to underage dirt bike riding. Unlike metropolitan centres, where paediatric fractures are more commonly associated with low-energy mechanisms, rural regions in Australia exhibit a unique injury profile. These findings offer an opportunity for public health interventions and region-specific injury prevention strategies. Critically, strengthening injury surveillance systems to inform policy development and measure the effectiveness of interventions over time, in the form of an accessible statewide or national rural trauma database, would greatly increase possibilities for the evaluation of trauma presentations in future analyses. In parallel, health system improvements, such as regular paediatric trauma training at the departmental level, the integration of specialist telehealth support, and streamlined direct transfer criteria to specialist centres are essential to reduce delays in definitive care and achieve best outcomes. By addressing these systemic gaps and risks, this study offers a foundation for multi-centre research and policy reform, ultimately aiming to enhance the trauma care, but more importantly to improve the cultural safety of children living in regional, rural, and remote communities.

## Figures and Tables

**Table 1:** Demographic data of paediatric trauma presentations included in this study [n (%)].

Gender	Hospital 1	Hospital 2	Total
Male	55 (48.7)	58 (51.3)	113 (75.3)
Female	15 (40.5)	22 (59.5)	37 (24.7)
**Age (years)**			
0–4	3 (33.3)	6 (66.7)	9 (6.0)
5–9	19 (50.0)	19 (50.0)	38 (25.3)
10–14	33 (51.6)	31 (48.4)	64 (42.7)
15–19	15 (38.5)	24 (61.5)	39 (26.0)
**Triage Category**			
Resuscitation-1	0 (0.0)	18 (100.0)	18 (12.0)
Emergency-2	70 (53.0)	62 (47.0)	132 (88.0)
**Mode of Arrival**			
Air Ambulance	1 (16.7)	5 (83.3)	6 (4.0)
Road Ambulance	33 (45.8)	39 (54.2)	72 (48.0)
Private Car	36 (50.7)	35 (49.3)	71 (47.3)

**Table 2:** More common upper and lower limb fractures [n (%)].

Upper Limb (Closed)	Proximal	Shaft	Distal	Total
Clavicle	2 (7.7)	20 (76.9)	4 (15.4)	26 (17.3)
Humerus	6 (42.9)	0 (0.0)	8 (57.1)	14 (9.3)
Radius	0 (0.0)	3 (18.8)	13 (81.2)	16 (10.7)
Ulna	1 (100.0)	0 (0.0)	0 (0.0)	1 (0.7)
Both Radius and Ulna	1 (3.9)	10 (38.5)	15 (57.7)	26 (17.3)
**Upper Limb (open)**				
Humerus	0 (0.0)	0 (0.0)	1 (100.0)	1 (0.7)
Both Radius and Ulna	0 (0.0)	3 (100.0)	0 (0.0)	3 (2.0)
**Lower Limb (closed)**				
Femur	2 (13.3)	11 (73.3)	2 (13.3)	15 (10.0)
Tibia	2 (20.0)	5 (50.0)	3 (30.0)	10 (6.7)
Fibula	0 (0.0)	2 (100.0)	0 (0.0)	2 (1.3)
Both Tibia and Fibula	2 (28.6)	2 (28.6)	3 (42.9)	7 (4.7)
**Lower Limb (open)**				
Tibia	0 (0.0)	1 (100.0)	0 (0.0)	1 (0.7)
Both Tibia and Fibula	0 (0.0)	2 (50.0)	2 (50.0)	4 (2.7)

**Table 3:** Less common “other” fractures (spine, pelvis, hand, and foot) [n (%)].

Spine	C-Spine	T-Spine	L-Spine	Sacrum	Total
	3 (27.3)	5 (45.5)	1 (9.1)	2 (18.2)	11 (7.3)
**Hand**	Prox Phalanx	Mid Phalanx	Open Phalanx		
	1 (16.7)	4 (66.7)	1 (16.7)		6 (4.0)
**Foot**	Ankle	Cuniform	Calcaneus	Navicular	
	1 (20.0)	1 (20.0)	2 (40.0)	1 (20.0)	5 (3.3)
**Pelvis**	Multiple				
	2 (100.0)				2 (1.3)

**Table 4:** Distribution of injuries by age group and mechanism of injury [n (%)].

Mechanism of Injury	0–4 Yrs	5–9 Yrs	10–14 Yrs	15–19 Yrs	Total
MBA	0 (0.0)	10 (26.3)	33 (51.6)	18 (46.2)	61 (40.7)
Falls related	4 (44.4)	8 (21.1)	10 (15.6)	6 (15.4)	28 (18.6)
MVA	2 (22.2)	3 (7.9)	6 (9.4)	8 (20.5)	19 (12.7)
Playground related	1 (11.1)	11 (29.0)	6 (9.4)	0 (0.0)	18 (12.0)
Equestrian related	0 (0.0)	2 (5.3)	6 (9.4)	0 (0.0)	8 (5.3)
Bicycle related	1 (11.1)	2 (5.3)	1 (1.6)	2 (5.1)	6 (4.0)
Crush Injury	1 (11.1)	0 (0.0)	1 (1.6)	2 (5.1)	4 (2.7)
Sports related	0 (0.0)	1 (2.6)	1 (1.6)	1 (2.6)	3 (2.0)
Aquatic Injury	0 (0.0)	1 (2.6)	0 (0.0)	2 (5.1)	3 (2.0)
**Total**	9	38	64	39	150

**Table 5:** Common mechanism of injury for each category of fracture presenting to both hospitals.

Fracture Category	Most Common Mechanism of Injury	Number n (%)
Spine	MBA	8 (72.7)
Humerus	=Playground related	4 (26.7)
	=Equestrian related	4 (26.7)
Radius	MBA	10 (62.5)
Radius + Ulna	Playground related	5 (17.2)
Ulna	nil	-
Clavicle	MBA	15 (57.7)
Femur	MBA	4 (26.7)
Tibia	MBA	4 (36.4)
Fibula	nil	-
Tibia + Fibula	MBA	5 (45.5)
Hand	nil	-
Foot	MBA	2 (40.0)
Pelvis	MVA	2 (100.0)

**Table 6:** Management of each respective category of fracture [n (%)].

Fracture Category	Non-op	CR	CR + KW	ORIF	IM Nail	Transfer Required
Spine	7 (63.6)	-	-	-	-	4 (36.4)
Humerus	6 (40.0)	-	5 (33.3)	3 (20.0)	1 (6.7)	-
Radius	4 (25.0)	8 (50.0)	2 (12.5)	2 (12.5)	-	-
Radius + Ulna	3 (10.4)	15 (51.7)	3 (10.4)	4 (13.8)	3 (10.4)	1 (3.5)
Ulna	-	1 (100.0)	-	-	-	-
Clavicle	25 (96.8)	-	-	1 (3.9)	-	-
Femur	7 (46.7)	-	-	-	8 (53.3)	-
Tibia	4 (36.4)	5 (45.5)	-	2 (18.2)	-	-
Tibia + Fibula	-	6 (54.5)	2 (18.2)	3 (27.3)	-	-
Fibula	2 (100.0)	-	-	-	-	-
Hand	7 (100.0)	-	-	-	-	-
Foot	4 (100.0)	-	-	2 (33.3)	-	-
Pelvis	1 (50.0)	-	-	-	-	1 (50.0)
**TOTAL**	**Non-op**	**Operative**				**Transfer Required**
	70 (46.7)	74 (49.3)				6 (4.0)

CR—Closed reduction; CR + KW—Closed reduction + K-wire fixation; ORIF—Open reduction, internal fixation; IM Nail—Intramedullary nail.

## Data Availability

All patient data associated with this study are securely stored in an encrypted database at the University of Sydney, located on the Camperdown campus. Access was restricted to authorised personnel via a VPN and a secured university faculty login, which securely connects to the university’s mainframe. Data cannot be transferred or stored on personal devices, ensuring compliance with privacy and security protocols. Patient data was used to search patient files on the NSW Health electronic medical records system to retrieve further information as required.
